# Collision Tumour of the Ampulla of Vater: Carcinoid and Adenocarcinoma

**DOI:** 10.1155/1997/46751

**Published:** 1997

**Authors:** I. M. Williams, N. W. Williams, D. Stock, M. E. Foster

**Affiliations:** Departments of Surgery and Pathology East Gtamorgan General Hospital Church Village Nr. Pontypridd Mid Gtamorgan CF38 1AB Barbados

## Abstract

Obstructive jaundice is most commonly due to luminal stones or lesions of the head of the pancreas and more rarely ampullary and primary common bile duct lesions. Obstruction due to lesions of the ampulla of Vater may be due to adenocarcinoma which has a significantly better long term prognosis than carcinomas located in the head of the pancreas. A case is presented where two tumours were identified at the ampulla of Vater of the resected specimen one an adenocarcinoma and the other a carcinoid tumour representing a collision tumour.


*HPB Surgery*, 1997, Vol. 10, pp. 241–244
Reprints available directly from the publisher
Photocopying permitted by license only

© 1997 OPA (Overseas Publishers Association)
Amsterdam B.V. Published in The Netherlands
by Harwood Academic Publishers
Printed in India

### *Case Report*

### Collision Tumour of the Ampulla of Vater: Carcinoid and Adenocarcinoma

I. M. WILLIAMS[a], N. W. WILLIAMS[b], D. STOCK[c] and M. E. FOSTER[d]

*Senior Surgical Registrar [a], Senior Registrar Pathology [b], Consultant Pathologist [c], Consultant Surgeon[d]*
*Departments of Surgery and Pathology, East Glamorgan General Hospital, Church Village, Nr. Pontypridd Mid Glamorgan, CF38 1AB*

*(Received 12 February 1996)*

**Obstructive jaundice is most commonly due to luminal stones or lesions of the head of the pancreas and more rarely ampullary and primary common bile duct lesions. Obstruction due to lesions of the ampulla of Vater may be due to adenocarcinoma which has a significantly better long term prognosis than carcinomas located in the head of the pancreas. A case is presented where two tumours were identified at the ampulla of Vater of the resected specimen one an adenocarcinoma and the other a carcinoid tumour representing a collision tumour.**

*Keywords:* Ampulla, Carcinoma, Carcinoid

### CASE REPORT

A 58 year old man presented with a six week history of increasing weight loss, anorexia and jaundice. Physical examination was unremarkable, but routine blood tests revealed a markedly elevated bilirubin and alkaline phosphatase with a normal coagulation screen and haemoglobin. An ultrasound scan was performed and showed dilated intra and extrahepatic bile ducts. An endoscopic retrograde cholangiopancreatography (ERCP) showed a dilated pancreatic duct and common bile duct but with no stones present. The distal common bile duct tapered and although no obstructive lesion was obvious biopsies were taken at the ampulla and a sphincterotomy was performed. A computerised tomogram indicated a dilated biliary system but no evidence of para aortic lymphadenopathy. The ampullary biopsy specimen underwent histological examination and this confirmed ampullary adenocarcinoma. In view of the fitness of the patient and no evidence of intra-abdominal spread of the tumour, he was prepared for a laparotomy and a Whipple's procedure was performed. The post-operative course was uneventful and he was discharged home fourteen days later.

### Histopathology

There were two adjacent tumours at the ampulla of Vater. There was an invasive moderately differentiated adenocarcinoma measuring 6 mm. It was seen to infiltrate into the muscle of the ampulla and into the submucosa of the duodenum and lower end of the common bile duct. Adjacent and just distal to this adenocarcinoma

Correspondence to: M. E. Foster, Dept. of Surgery, East Glamorgan Hospital, Church Village, Mid Glamorgan.

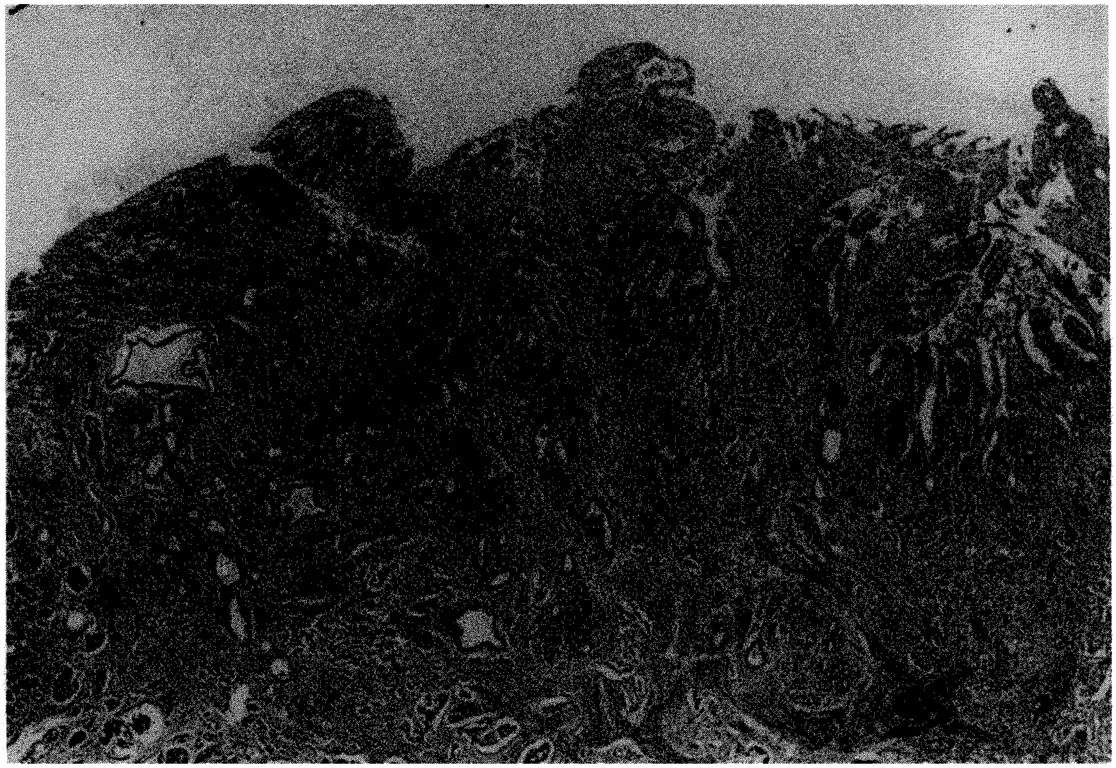

FIGURE 1 The ampulla of Vater with an infiltrating carcinoid tumour (left and centre) and an adenocarcinoma (bottom right). (H and E, x25).

was a 6 mm carcinoid tumour composed of islands and cords of relatively regular, argyrophilic cells with granular cytoplasm. The carcinoid tumour infiltrated the ampulla and duodenal submucosa and lymphatic permeation was seen (Fig. 1).

Local excision of the adenocarcinoma and carcinoid tumour appeared complete. A lymph node in the pancreaticoduodenal groove was replaced by carcinoid tumour. A small deposit of adenocarcinoma was also present in this node. The appearances of the two morphologically different tumours at the ampulla of Vater were in keeping with a collision tumour. This means two separate tumours occuring at the same site in the same patient, rather than a single tumour showing two patterns of differentiation.

Immunocytochemical studies showed positive staining of the carcinoid component with antibodies directed against serotonin, chromogranin A and gastrin (Fig. 2). There was negative staining of the adenocarcinomatous and carcinoid components with antibodies directed against insulin, vasoactive intestinal peptide (VIP), glucagon, somatostatin, pancreatic polypeptide and bombesin. The adenocarcinomatous component stained positively with antibodies directed against carcino-embryonic antigen (CEA).

### DISCUSSION

The simultaneous occurrence of carcinoid tumour and adenocarcinoma of the ampulla of Vater, to the best of our knowledge, has not been previously reported. The 5 year long term survival rates for adenocarcinoma of the ampulla are superior to the relatively poor survival rates for carcinomas of the head of the pancreas [1–3]. Reports of long term survival following surgical resection of

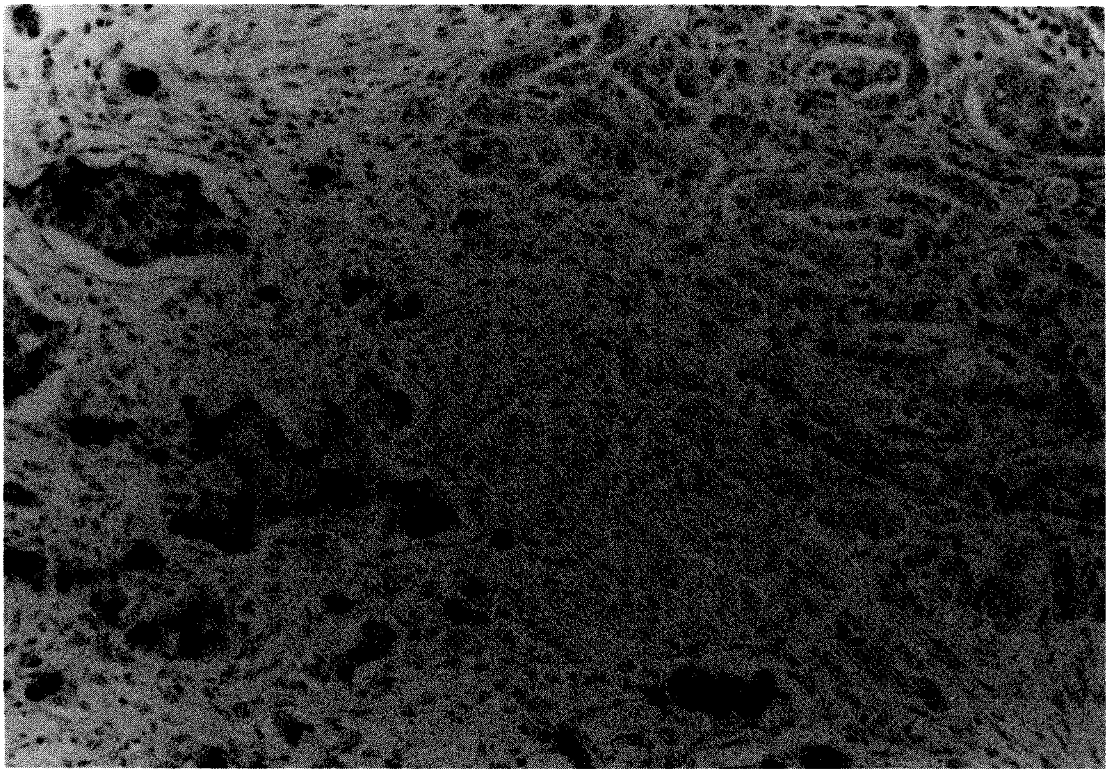

FIGURE 2 The 'collision point' of the carcinoid tumour (left) with the adenocarcinoma (right). There is focal immunocytochemical staining of the carcinoid tumour cells using antibodies directed against gastrin (DAB reaction product, gastrin x200).

carcinoid tumours of the ampulla is significantly better, with some describing up to 90% of patients surviving five years post surgery [4].

Clinicopathological staging of carcinoid tumours was initially based on the behaviour of the commoner small bowel carcinoids. Tumours less than 2.5 cm in diameter were classified as superficial, whilst those of larger diameter were considered deeply invasive [5]. This classification system demonstrated less than 1% of the superficial carcinoid tumours has nodal metastases. This compared to over 80% of the deeply invasive carcinoid tumours. There is evidence carcinoid tumours of the ampulla of Vater behave differently from those arising in the small bowel. A study by Ricci of 27 patients with carcinoid tumour of the ampulla of Vater demonstrated that 53% with carcinoid tumours less than 2 cm in diameter were found on histological examination to have node positive disease [6]. The diameter of the carcinoid tumour at the ampulla of Vater seems to be unimportant as a predictor for the metastatic potential of the lesion. For this reason radical pancreaticoduodenectomy has been proposed as the optimum treatment modality for carcinoid tumours rather than local ampullectomy [7].

The carcinoid tumour resected in this case is one of the smallest recorded at 6 mm. Despite this, nodal metastases were detected, confirming the unpredictable behaviour of these tumours.

### References

[1] Hayes, D.H., Bolton, J.S., Willis, G.W. and Bowen, J.C. (1987). Carcinoma of the ampulla of Vater. *Ann Surg.*, **206**, 572–577.

[2] Knox, R.A. and Kingston, R.D. (1986). Carcinoma of the ampulla of Vater. *Br J Surg.*, **73**, 72–73.

[3] Sperti, C., Pasquali, C., Piccoli, A., Sernagiotto, C. and Pedrazzoli, S. (1994). Radical resection for ampullary carcinoma: long term results. *Br J Surg.*, **81**, 668–671.

[4] Hatzitheoklitos, E., Buchler, M.W., Freiss, H., Poch, B., Ebert, M., Mohr, W., Imaizumi, T. and Beger, H.G. (1994). *Carcinoid of the ampulla of Vater. Cancer*, **73**, 1580–1588.
[5] Hajdu, S.I., Winawer, S.J. and Myers, W.P. (1974). Carcinoid tumours: a study of 204 cases. *Am J Clin Pathol.*, **61**, 521–528.
[6] Ricci, J.L. (1993). Carcinoid of the ampulla of Vater. *Cancer*, **71**, 686–690.
[7] Asbun, H.J., Rossi, R.L. and Munson, L. (1993). Local resection for ampullary tumours. *Arch Surg*, **128**, 515–520.

### COMMENTARY

This manuscript points out an important finding the possibility of coexistence of an endocrine and an epithelial neoplasm. While the pathologic significance of two lesions is not totally clear in this paper as written, the coexistence brings up several good questions: 1) Which would determine the course of the patient? Patient died 24 months later from disseminated adenocarcinoma. Of course, the staging of the two tumors would probably be the answer. 2) Is there anything prognostic about the coexistence of the two lesions? Maybe.

And, 3) could there be some significance in terms of etiology? This latter question is interesting, but not at the present time answerable. However, the incidence of the coexistence of the two lesions is likely higher than appreciated. For example, endocrine micronests were found near the papillae in 52% of 78 autopsy and 117 surgical specimens in one study [1]. The coexistence of these lesions has been reported before [2]. And both adenocarcinoma of the stomach and endocrine appear after long-standing absence of acid or long term omeprazole therapy. Could there be etiologic link. Let's be prepared for that possibility, particularly since somatostatin or its receptors have a high incidence of existence in ampullary lesions in general [3].

This report is only observational but could be making an important observation. Let's see what others report over the next few years.

William C. Meyers, M.D.
Professor and Chief of Surgery
University of Massachusetts

### *References*

[1] Noda, Y., Watanabe, J., Iida, M., Narisawa, R., Kurosaki, I., Iwafuchi, M., Satoh, M. and Ajioka, Y. (1992). Histologic follow-up of ampullary adenomas in patients with familial adenomatosis coli. *Cancer*, **70**(7), 1847–56.
[2] Misonou, J., Kanda, M., Kitagawa, T., Ota, T., Muto, E., Nenohi, M. and Atsuts, T. (1990). A case of coexisting malignant carcinoid tumor and adenocarcinoma in the papailla of Vater. *Gastroenterologia Japonica.*, **25**(5), 630–5.
[3] Ahearne, P., Baillie, J. and Meyers, W. Unusual tumors of the ampulla of Vater. In Sarr M, *et. al.* (eds): The Pancreas. Blackwell Scientific Publishers. (In press).

